# The association between parity and spontaneous preterm birth: a population based study

**DOI:** 10.1186/s12884-020-02940-w

**Published:** 2020-04-21

**Authors:** Bouchra Koullali, Maud D. van Zijl, Brenda M. Kazemier, Martijn A. Oudijk, Ben W. J. Mol, Eva Pajkrt, Anita C. J. Ravelli

**Affiliations:** 1 Department of Obstetrics and Gynecology, Amsterdam UMC, University of Amsterdam, Meibergdreef 9, 1105 AZ Amsterdam, The Netherlands; 2 Department of Obstetrics and Gynecology, School of Medicine, Monash University, Melbourne, Australia; 3 Department of Medical Informatics, Amsterdam UMC, University of Amsterdam, Meibergdreef 9, Amsterdam, The Netherlands

**Keywords:** Spontaneous preterm birth, Parity, Nulliparity, Risk factors

## Abstract

**Background:**

Preterm birth is the leading cause of perinatal mortality and neonatal morbidity worldwide. Many factors have been associated with preterm birth, including parity. The aim of the present study was to investigate associations between parity and risk of spontaneous preterm birth.

**Methods:**

We conducted a retrospective study including live singleton births (≥22 weeks) of women with a first, second, third, fourth or fifth pregnancy in The Netherlands from 2010 through 2014. Our primary outcome was risk of spontaneous preterm birth < 37 weeks. Secondary outcomes were spontaneous preterm birth < 32 and < 28 weeks.

**Results:**

We studied 802,119 pregnancies, including 30,237 pregnancies that ended spontaneously < 37 weeks. We identified an increased risk for spontaneous preterm birth < 37 weeks in nulliparous women (OR 1.95, 95% CI 1.89–2.00) and women in their fifth pregnancy (OR 1.26, 95% CI 1.13–1.41) compared to women in their second pregnancy. Similar results were seen for spontaneous preterm birth < 32 and < 28 weeks.

**Conclusion:**

Our data show an independent association between nulliparity and spontaneous preterm birth < 37, < 32 and < 28 weeks. Furthermore, we observed an increased risk for spontaneous preterm birth in women in their fifth pregnancy, with highest risk for preterm birth at early gestational age.

## Background

Preterm birth, defined as birth before 37 weeks of gestation, is the leading cause of perinatal mortality and neonatal morbidity worldwide, mostly due to respiratory immaturity, intracranial hemorrhages and infections [1, 2]. Morbidity and mortality rates increase with decreasing gestational age [3]. Fifteen million children are born preterm worldwide each year, of which almost two and a half million children are born before 32 weeks of gestation [4].

Preterm birth is considered a syndrome that can be initiated by multiple mechanisms such as intrauterine infection and inflammation, uteroplacental ischemia and hemorrhage, uterine overdistension, cervical insufficiency, hormonal disorders, and other immunologically mediated processes [5]. Defining maternal risk factors for preterm birth in epidemiological studies can provide important insights into mechanisms that lead to preterm birth and help to identify women at risk. This can lead to the introduction of risk-specific treatment and counseling [6].

There are many maternal characteristics that have been associated with preterm birth, including demographic characteristics (i.e. low socioeconomic status), low or high body-mass index (BMI), smoking and a previous preterm birth [6, 7]. Parity is another factor associated with preterm birth, with the highest rates reported in nulliparous women and the lowest rates reported in second births [8]. Studies on the association between high parity and adverse pregnancy outcomes show conflicting results. A number of studies did report an association between high parity and adverse pregnancy outcomes [9, 10]. In contrast, other studies state that, under satisfactory socioeconomic and health care conditions, high parity should not be considered as a risk factor for adverse pregnancy outcomes [11]. A systematic review from 2010 shows that grand multiparity and great grand multiparity were not associated with increased risk of preterm birth [12].

The principal aim of the present study was to investigate associations between parity and risk of spontaneous preterm birth, assessing first, second, third, fourth and fifth pregnancies, using a large population-based study.

## Methods

### Dataset

This study was based on data from the Netherlands Perinatal Registry (PERINED). This database is a population based registry that covers approximately 97% of all deliveries in The Netherlands and contains information on deliveries at ≥22 weeks of gestation and birth weight of ≥500 g. Furthermore, all admissions to the neonatology care unit are registered until 28 days after birth. The perinatal database is obtained by a validated linkage of 3 different registries: the midwifery registry (LVR1), the obstetrics registry (LVR2), and the neonatology registry (LNR) of hospital admissions of new-born infants [13, 14]. It is used primarily for an annual assessment of the quality indicators of obstetric care.

### Ethical approval

The data in the perinatal registry are anonymous; therefore, ethical approval was not mandatory under Dutch law. The Netherlands Perinatal Registry gave their approval for the use of their data for this study (approval no. 17.34).

### Inclusion and exclusion criteria

We studied singleton first, second, third, fourth and fifth pregnancies (P0 through P4) resulting in delivery between 22 and 43 weeks of gestation in the 5-year period from 2010 through 2014. We excluded multiple pregnancies and pregnancies that were complicated by congenital abnormalities or stillbirth.

### Outcome measures

Our primary outcome was risk of spontaneous preterm birth < 37 weeks of gestation per parity. Other outcome variables were spontaneous preterm birth < 32 and < 28 weeks. We performed additional analyses for the outcome late spontaneous preterm birth between 34 and 37 weeks to assess pregnancies in women that were not offered additional screening or treatment to prevent recurrent preterm birth. The PERINED registry contains data on whether a delivery started spontaneous (i.e., with spontaneous rupture of the membranes or contractions) or iatrogenic (i.e., planned Caesarean section or induction of labor).

### Statistical analysis

To estimate the effect of parity on spontaneous preterm birth < 37, < 32 and < 28 weeks (and between 34 and 37 weeks), we used a univariate logistic regression model and expressed the effect estimates as odds ratios (OR) and corresponding 95% confidence intervals (CI). We used multivariate logistic regression analysis to adjust for the most common known risk factors for preterm birth that were available in the national perinatal registry that we used for our study. The chosen variables were based on previous studies about risk factors for (spontaneous) preterm birth [6, 15, 16]. First, we adjusted for possible maternal confounders (**correction model A**) including maternal age (< 20 years, ≥40 years and continuous), non-White ethnicity, low socioeconomic status (SES), and, in multiparous women, a prior preterm birth. Additional analysis were performed to adjust for potentially mediating factors occurring in the pathway between the independent (parity) and dependent (spontaneous preterm birth) variables (**correction model B**). Correction model B included the maternal confounders as in model A and in addition artificial reproductive techniques (ART), male fetal gender, hypertension, preeclampsia and small for gestational age (SGA) < p10. All variables were extracted from PERINED, including SES which was based on the 4 digit postal code of the woman’s home address. SES was divided into low (< 25%), middle (25–75%) and high (> 75%) status.

In multiparous women (P1 through P4), we used the Cochran-Armitage Trend Test to test for a trend in parity on the incidence of spontaneous preterm birth < 37, < 32 and < 28 weeks (and between 34 and 37 weeks). The data were analyzed with the SAS statistical software package (version 9.3; SAS Institute Inc., Cary, NC).

## Results

We identified 837,226 singleton pregnancies of women who delivered ≥22 weeks of gestation from 2010 through 2014. We excluded pregnancies complicated by stillbirth (3118, [0.37%]) or congenital abnormalities (25,444, [3.04%]). The total of first, second, third, fourth and fifth pregnancies (P0 through P4) with complete follow-up data was 802,119, of which 30,237 (3.8%) were spontaneous preterm births < 37 weeks of gestation. The proportion of pregnancies per parity was 45.8% (*n* = 367,676) in P0, 36.1% (*n* = 289,391) in P1, 13.1% (*n* = 105,014) in P2, 3.8% (*n* = 30,585) in P3 and 1.2% (*n* = 9453) in P4 (Table 1).

**Table 1:** Comparison of maternal and pregnancy characteristics and outcomes between the different parity groups

	*Total*	*P0*	*P1*	*P2*	*P3*	*P4*	P-value*
*Number of subjects (%)*	802,119 (100)	367,676 (45.8)	289,391 (36.1)	105,014 (13.1)	30,585 (3.8)	9453 (1.2)	NA
*Mean GA in weeks (SD)*	39.1 (1.8)	39.08 (2.0)	39.18 (1.6)	39.15 (1.7)	39.03 (1.8)	38.94 (2.0)	NA
*Mean maternal age (SD)*	30.8 (4.8)	29.25 (4.9)	31.53 (4.4)	32.98 (4.3)	34.03 (4.4)	35.03 (4.5)	<.0001
*Maternal age < 20 yrs (%)*	10,201 (1.3)	9230 (2.5)	902 (0.3)	61 (0.1)	7 (0.02)	1 (0.01)	<.0001
*Maternal age ≥ 40 yrs (%)*	24,797 (3.1)	6891 (1.9)	8505 (2.9)	5310 (5.1)	2785 (9.1)	1306 (13.8)	<.0001
*Non-White (%)*	152,899 (19.1)	63,754 (17.3)	49,303 (17.0)	25,427 (24.2)	10,650 (34.8)	3765 (39.8)	<.0001
*Low SES (%)*	197,136 (24.6)	91,433 (24.9)	65,885 (22.8)	27,006 (25.7)	9602 (31.4)	3210 (34.0)	<.0001
*Prior PTB (%)*	6858 (0.9)	0	4208 (1.5)	1825 (1.7)	623 (2.0)	202 (2.1)	<.0001
*ART (%)*	29,763 (3.7)	19,838 (5.4)	8148 (2.8)	1436 (1.4)	276 (0.9)	65 (0.7)	<.0001
*Male fetal gender (%)*	409,097 (51.0)	187,681 (51.1)	147,586 (51.0)	53,361 (50.8)	15,588 (51.0)	4881 (51.63)	0.6184
*Hypertension and preeclampsia (%)*	70,745 (8.8)	43,656 (11.9)	18,610 (6.4)	5972 (5.7)	1869 (6.1)	638 (6.8)	<.0001
*SGA < p10 (%)*	67,744 (8.5)	30,252 (8.2)	25,431 (8.8)	8493 (8.1)	2724 (8.9)	844 (8.9)	0.0002
*Total PTB*	43,653 (5.4)	25.483 (6.9)	11,769 (4.1)	4353 (4.2)	1491 (4.9)	557 (5.9)	NA
*Spontaneous PTB*	30,237 (3.8)	18,171 (4.9)	8065 (2.8)	2761 (2.6)	893 (2.9)	347 (3.7)	NA
*Iatrogenic PTB*	13,416 (1.7)	7312 (2.0)	3704 (1.3)	1592 (1.5)	598 (2.0)	210 (2.2)	NA
*Previous PTB in women with PTB*	1446 (8.0)	NA	942 (8.0)	343 (7.9)	121 (8.1)	40 (7.2)	NA

Abbreviations: *GA* gestational age; *SD* standard deviation; *yrs* years; *SES* socio-economic status; *PTB* preterm birth; *ART* artificial reproductive techniques; *SGA* small for gestational age

### Maternal and pregnancy characteristics per parity

The proportion of pregnancies per parity plus the maternal and pregnancy characteristics of the parity groups are presented in Table 1. The mean maternal age increased with higher parity from 29.25 years in P0 to 35.03 years in P4 (*p* < .0001). The percentage of non-white ethnicity increased with higher parity, 17.3% non-White in P0 compared to 39.8% in P4 (p < .0001). Also the percentage of women with a low SES increased with higher parity, 24.9% low-SES in P0 increasing to 34.0% in P4 (p < .0001). Hypertension and preeclampsia occurred more often in nulliparous women while these rates remained relatively stable in multiparous women (Table 1).

### Preterm birth incidence by parity

**Fig. 1:**
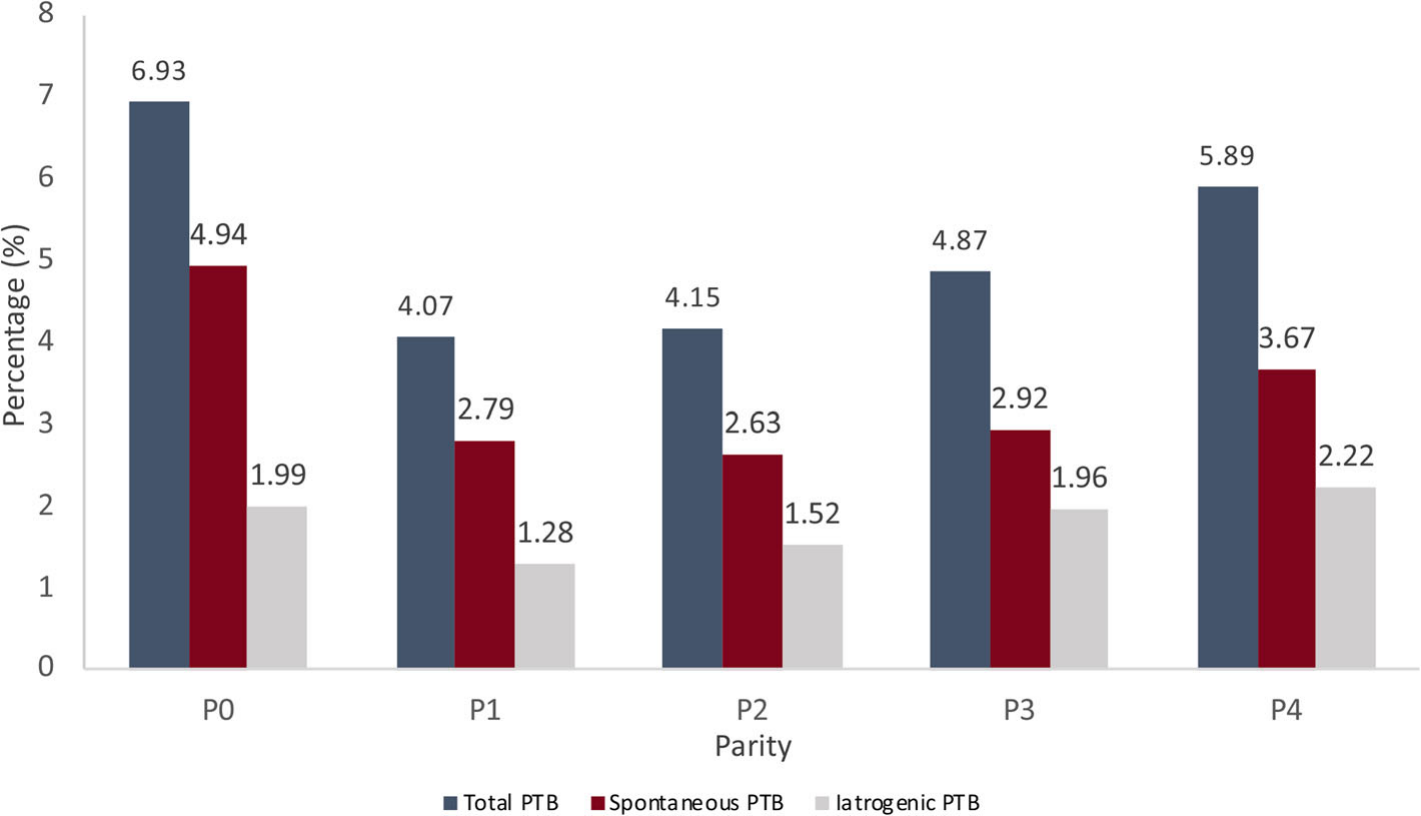
Incidence rates of overall preterm birth and stratified for spontaneous and iatrogenic preterm birth for women in their first (P0), second (P1), third (P2), fourth (P3) and fifth (P4) pregnancy from 2010 through 2014 in The Netherlands. Abbreviations: PTB, preterm birth

The overall incidence of preterm birth < 37 weeks of gestation among singletons without congenital anomalies was 5.4% in The Netherlands during the 5 year study period. Rates of spontaneous and iatrogenic preterm birth < 37 weeks of gestation were 3.8 and 1.7%, respectively (Table 1). The incidence of total, spontaneous and iatrogenic preterm birth stratified for parity are presented in Fig. 1 and Table 1. The highest incidence of spontaneous preterm birth was observed among nulliparous women (P0, 4.9%) and women in their fifth pregnancy (P4, 3.7%) (Fig. 1 and Table 1). In addition, among the 18,170 women in their second, third, fourth or fifth pregnancy who had a preterm birth, 8% (*n* = 1446 out of 18,170 women) had a prior preterm birth and 92% were new preterm births (Table 1). These percentages per parity were 8.0% for P1, 7.9% for P2, 8.1% for P3 and 7.2% for P4 (Table 1).

### Parity and risk of spontaneous preterm birth by gestational age

**Fig. 2:**
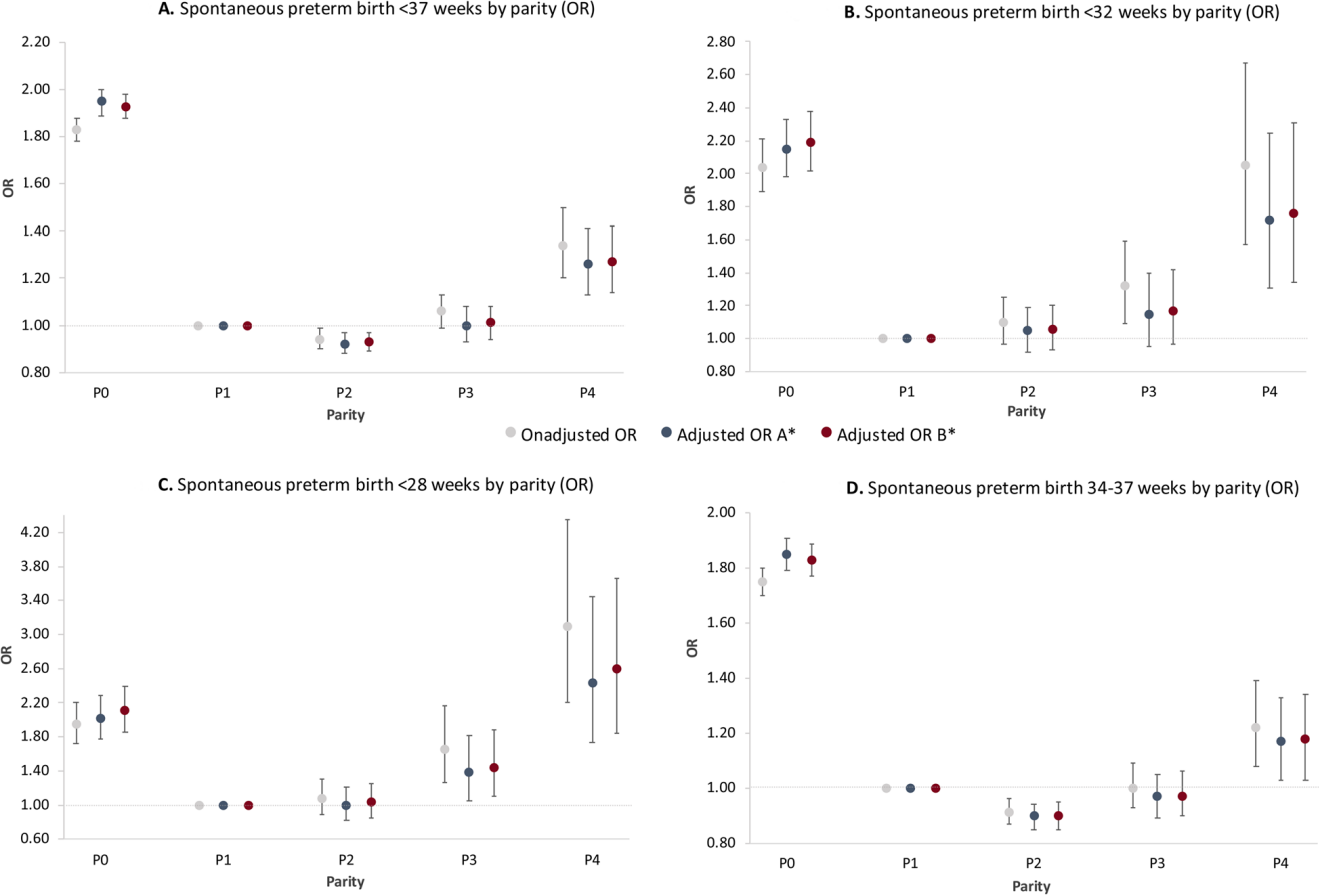
Odds ratio’s for spontaneous preterm birth (**a**) < 37 weeks, (**b**) < 32 weeks, (**c**) < 28 weeks and between (**d**) 34–37 weeks of gestation per parity. Abbreviations: OR, odds ratio. ^*^Correction **model A**: adjusted for maternal age (< 20 years, ≥40 years and continuous), non-White ethnicity, low socioeconomic status, and a prior preterm birth. Correction **model B**: adjusted for A and artificial reproductive techniques, male fetal gender, hypertension, and small for gestational age < p10

Spontaneous preterm birth risks by gestational age were examined for parity. We used women in their second pregnancy (P1) as reference; the results are demonstrated in Table 2 and Fig. 2.

**Table 2:** Relation between parity and spontaneous preterm birth < 37, < 32, < 28 and between 34 and 37 weeks of gestation

	*Unadjusted OR (95% CI)*	A^*^: Adjusted OR (95% CI)	B^**^: Adjusted OR (95% CI)
PTB < 37 weeks			
P0	1.83 (1.78–1.88)	1.95 (1.89–2.00)	1.93 (1.88–1.98)
P1 (ref)	1.0	1.0	1.0
P2	0.94 (0.90–0.99)	0.92 (0.88–0.97)	0.93 (0.89–0.97)
P3	1.06 (0.99–1.13)	1.00 (0.93–1.08)	1.01 (0.94–1.08)
P4	1.34 (1.20–1.50)	1.26 (1.13–1.41)	1.27 (1.14–1.42)
PTB < 32 weeks			
P0	2.04 (1.89–2.21)	2.15 (1.98–2.33)	2.19 (2.02–2.38)
P1 (ref)	1.0	1.0	1.0
P2	1.10 (0.97–1.25)	1.05 (0.92–1.19)	1.06 (0.93–1.20)
P3	1.32 (1.09–1.59)	1.15 (0.95–1.40)	1.17 (0.97–1.42)
P4	2.05 (1.57–2.67)	1.72 (1.31–2.25)	1.76 (1.34–2.31)
PTB < 28 weeks			
P0	1.95 (1.72–2.20)	2.02 (1.78–2.29)	2..11 (1.86–2.39)
P1 (ref)	1.0	1.0	1.0
P2	1.07 (0.89–1.30)	1.00 (0.82–1.21)	1.03 (0.85–1.25)
P3	1.65 (1.27–2.16)	1.38 (1.05–1.81)	1.44 (1.10–1.88)
P4	3.10 (2.21–4.35)	2.44 (1.73–3.45)	2.59 (1.84–3.66)
PTB 34–37 weeks			
P0	1.75 (1.70–1.80)	1.85 (1.79–1.91)	1.83 (1.77–1.89)
P1 (ref)	1.0	1.0	1.0
P2	0.91 (0.87–0.96)	0.90 (0.85–0.94)	0.90 (0.85–0.95)
P3	1.00 (0.93–1.09)	0.97 (0.89–1.05)	0.97 (0.90–1.06)
P4	1.22 (1.08–1.39)	1.17 (1.03–1.33)	1.18 (1.03–1.34)

Abbreviations: *OR* odds ratio; *CI* confidence interval; *PTB* preterm birth

^*^Correction **model A**: Adjusted for maternal age (< 20 years, ≥40 years and continuous), non-White ethnicity, low socioeconomic status, and a prior preterm birth

^**^Correction **model B**: Adjusted for A and artificial reproductive techniques, male fetal gender, hypertension, preeclampsia, and small for gestational age < p10

Both nulliparous women and women in their fifth pregnancy had the highest risk for all preterm birth outcomes. Preterm birth risk in nulliparous women slightly increased after adjusting for confounders compared to the unadjusted risk, whereas in women in their fifth pregnancy the risk slightly decreased after correcting for the same confounders (Table 2 and Fig. 2).

For spontaneous preterm birth < 37 weeks we observed the highest risk in nulliparous women (OR 1.95, 95% CI 1.89–2.00) and women in their fifth pregnancy (OR 1.26, 95 CI 1.13–1.41) (Fig. 2a). For spontaneous preterm birth < 32 weeks, nulliparous women had the highest risk (OR 2.15, 95% CI 1.98–2.3) followed by women in their fifth pregnancy (OR 1.72, 95% CI 1.31–2.25) (Fig. 2b). Although we observed an increased risk for spontaneous preterm birth < 32 weeks in women in their fourth pregnancy, no effect was seen after adjusting for confounders (OR 1.15, 95% CI 0.95–1.40). The risk for spontaneous preterm birth < 28 weeks was highest in women in their fifth pregnancy (OR 2.44, 95% CI 1.73–3.45), followed by nulliparous women (OR 2.02, 95% CI 1.78–2.29) and women in their fourth pregnancy (OR 1.38, 95% CI 1.05–1.81) (Fig. 2c). Women in their first pregnancy had the highest risk for a spontaneous preterm birth between 34 and 37 weeks of gestation (OR 1.85, 95% CI 1.79–2.29) followed by women in their fifth pregnancy (OR 1.17, 95% CI 1.03–1.33) (Fig. 2d). We did not observe significant differences between results obtained from **model A** and **model B** in all parity groups and outcomes (Table 2).

### Trend in incidence of spontaneous preterm birth in multiparous women

After exclusion of nulliparous women, we observed an increase in incidence of spontaneous preterm birth < 37 weeks (*p* = 0.0178), < 32 weeks (*p* < .0001) and < 28 weeks (p < .0001), with increasing parity in multiparous women (Table 3). This trend was in line with our observation of increasing odds ratio’s in multiparous women for all three outcomes. No trend was seen in spontaneous preterm birth between 34 and 37 weeks (Table 3).

**Table 3:** Trend test in spontaneous preterm birth incidence rates in multiparous women

	*P1*	*P2*	*P3*	*P4*	P-value*
*Spontaneous PTB*					
*< 37 weeks*	8065 (2.8%)	2761 (2.6%)	893 (2.9%)	347 (3.7%)	0.0178
*< 32 weeks*	873 (0.3%)	349 (0.3%)	121 (0.4%)	58 (0.6%)	<.0001
*< 28 weeks*	367 (0.1%)	142 (0.1%)	64 (0.2%)	37 (0.4%)	<.0001
*34–37 weeks*	6406 (2.2%)	2118 2.0%)	675 (2.2%)	251 (2.7%)	NS

Abbreviations: *PTB* preterm birth

* Two-sided Cochran-Armitage Trend Test

## Discussion

In this nationwide retrospective study we found that nulliparity (P0) was independently associated with an overall increased risk for spontaneous preterm birth compared to women in their second pregnancy (P1). We also observed an increase in incidence of spontaneous preterm birth < 37, < 32 and < 28 weeks with higher parity in multiparous women, with highest risk for spontaneous preterm birth < 28 weeks in women in their fifth pregnancy.

The association between nulliparity and spontaneous preterm birth is supported by other studies [17, 18]. Our study also finds an association between high parity and spontaneous preterm birth. Previous studies mostly assessed the effect of (high) parity in the context of advanced maternal age [19] or state that the effect of parity is influenced by socioeconomic and health care conditions [11]. More studies have been conducted to assess the association between parity and adverse pregnancy outcomes, however, these studies do not assess preterm birth as a primary outcome [9, 10].

The conflicting results of the different studies point to the complexity of the association between possible risk factors, including parity, and spontaneous preterm birth. It also highlights the possible influence of factors that contribute to a higher risk of spontaneous preterm birth, such as ethnicity and socio-economic status. However, in the current study we found an association between high parity and spontaneous preterm birth while adjusting for established risk factors such as ethnicity and socio-economic status. This possibly points to other factors that may contribute to a higher risk of spontaneous preterm birth. One of the factors that may play a role could be a damaged cervix. The cervix plays an important role in maintaining pregnancy. It is well known that damage to the cervix, for instance by dilatation and curettage or loop excisions of the cervix for premalignant lesions, contributes to a higher risk of spontaneous preterm birth [20]. The risk of such procedures being performed is higher in women at higher age or parity, which may be an explanation for the association of parity and spontaneous preterm birth we found.

The overall risk for spontaneous preterm birth was significantly increased in nulliparous women compared to women in their second pregnancy, including the risk of birth between 34 and 37 weeks. According to the national prevention of preterm birth protocol in The Netherlands, women with a prior spontaneous preterm birth between 34 and 37 weeks are not offered additional screening or treatment (such as administration of progesterone, pessary or cerclage, and cervical length screening or bacterial vaginosis screening) and receive similar obstetric care as women without a prior preterm birth [21]. Also, it is unlikely that these treatment effects can explain these differences.

Although our results show that nulliparity and high parity is associated with an increased risk for spontaneous preterm birth, we observed remarkable differences between the association with nulliparity compared to high parity. While the risk of spontaneous preterm birth < 37, < 32 and < 28 weeks in nulliparous women is relatively similar, women in their fourth and women in their fifth pregnancy have a particularly high risk of spontaneous preterm birth occurring at early gestational age (Table 2).

We observed that odds ratios in nulliparous women increased after adjusting for confounders whereas odds ratio’s in multiparous women decreased after adjusting. These data point to differences in the effect of established confounders on spontaneous preterm birth between different parity groups. This is in line with the significant differences we observed in the confounders low and high maternal age, non-White ethnicity and low socio-economic status between nulliparous and multiparous women.

### Strengths and limitations of this study

The strengths of this study include the high quality of data in the PERINED registry which covers approximately 97% of all deliveries in the Netherlands. We were able to study a large recent set of pregnancies (*n* = 802,119), including first, second, third, fourth and fifth pregnancies, and 30,237 spontaneous preterm births < 37 weeks of gestation.

Multiple epidemiologic studies have reported associations of nulliparous women with increased risk of preterm birth [9, 18, 22–24]. Yet, in many of these studies, parity has been categorized as nulliparous and multiparous, with women with their second pregnancy often grouped in with those of higher-order parity. In our study, we evaluated the effect per parity separately which allowed us to identify the increased risk in both nulliparous women and women with higher parity.

Unfortunately, due to low reporting within the perinatal database, we were not able to correct for smoking during pregnancy and maternal body mass index (BMI) in our analyses. The general incidence of smoking in The Netherlands is 22.4% in the population > 18 years old, 19.2% of all women are smokers [25]. The incidence of smoking in pregnant women in The Netherlands is 7.4% [26]. The general incidence of obesity in The Netherlands is 50.2% in the population > 18 years, of all women 47.2% has obesity (30.4% has moderate obesity and 16.9 has severe obesity) [27]. Smoking and very low or very high maternal BMI are known risk factors for spontaneous preterm birth [28, 29]. This may have influenced our results. Because we corrected for low socio-economic status in our analyses, and it is known that low socio-economic status is strongly correlated to both smoking and maternal obesity, we do not think that this issue of missing adjustment factors has influenced our results to a large degree. In addition to smoking and BMI, we were not able to correct for other potential risk factors that contribute to the risk of preterm birth, such as polyhydramnios, intra-uterine infection, single marital status, short interpregnancy interval (< 6 months) and specific maternal diseases (uterus anomaly, cervical excision procedures, maternal surgery during pregnancy, depression) [6].

Pregnancies ending < 22 weeks were not included in our national database. Although we corrected for a prior preterm birth, which was available in our dataset, we did not have information on multiple occurrence nor severity of the prior preterm birth. Because no longitudinal linked obstetric database was available, pregnancies could not be related to the level of the individual woman in this study. We therefore could not identify women that were included multiple times due to multiple pregnancies between 2010 and 2014 which may have influenced our results.

## Conclusion

Our findings indicate that high parity, as well as nulliparity, is involved as a risk factor in the complex pathways that lead to spontaneous preterm birth. These results highlight the importance of the effect of parity on spontaneous preterm birth and may assist in preterm birth risk stratification and counseling.

## Data Availability

This study was based on data from the Netherlands Perinatal Registry (PERINED). This database is a population based registry that is not publicly accessible. Approval from PERINED is acquired.

## Abbreviations

ART: Artificial reproductive techniques
GA: Gestational age
OR: Odds ratio
PTB: PRETERM birth
SD: Standard deviation
SES: Socio-economic status
SGA: Small for gestational age
